# The first Choosing Wisely Africa conference: a roadmap to value-based cancer care in Africa (16th December 2022, Senegal)

**DOI:** 10.3332/ecancer.2022.1517

**Published:** 2023-03-06

**Authors:** Melinda Mushonga, Omar Abdihamid, Christian Ntizimira, Gad Murenzi, Sidy Ka, Nazik Hammad, Fidel Rubagumya

**Affiliations:** 1 Sunnybrook Health Sciences, Odette Cancer Centre, University of Toronto, Toronto, ON M4N 3M5, Canada; 2 Department of Oncology, Faculty of Medicine and Health Sciences, University of Zimbabwe, Harare, Zimbabwe; 3 The Garissa Cancer Center, Garissa County Referral Hospital, Garissa, Kenya; 4 African Center for Research on End-of-Life Care, Kigali, Rwanda; 5 Einstein-Rwanda Research and Capacity Building Program, Research for Development (RD Rwanda) and Rwanda Military Hospital, Kigali, Rwanda; 6 Cheikh Anta Diop University, Dakar 10700, Senegal; 7 Departments of Oncology, Queen’s University, Kingston, ON K7L 3N6, Canada; 8 Department of Oncology and Department of Research, Rwanda Military Hospital, Kigali, Rwanda; 9 Faculty of Medicine, University of Rwanda, Kigali, Rwanda

**Keywords:** Choosing Wisely Africa inaugural conference report, updates and recommendations, Senegal 2022

## Abstract

The Choosing Wisely campaign was formally launched in 2012 and a decade later, the inaugural Choosing Wisely Africa conference was held in Dakar, Senegal on 16 December 2022 supported by ecancer. Academic partners included Ministere de la Sante et de I’Action Sociale, Senegalese Association of Palliative Care, Federation Internationale des Soins Palliatifs, Universite Cheikh Anta diop de Dakar, Societe Senegalaise de Cancerologie and King’s College London. There were around 70 delegates attending in person mostly from Senegal and a further 30 joining virtually. Ten speakers gave insight into Choosing Wisely from an African perspective and Dr’s Fabio Moraes and Frederic Ivan Ting shared the Choosing Wisely experience from Brazil and the Philippines, respectively. This report therefore shares the highlights of the first Choosing Wisely Africa conference guided by topics discussed.

## Background of Choosing Wisely Africa (CWA)

Choosing Wisely is an American Board of Internal Medicine initiative, which aims to advance better decision-making about health care delivery by promoting discussion between clinicians and patients about care that is supported by evidence, not duplicative of other tests or procedures already received, free from harm and truly necessary. Since its inception, it has become relevant across all income settings as it promotes equitable use of health care resources. Therefore, a comprehensive background of the inception of the Choosing Wisely campaign leading up to the development of CWA guidelines was shared by most speakers including its relevance in Africa from a resource perspective where the cancer burden is rising against a background of limited resources and workforce in some regions. Dr Fabio Moraes and Dr Frederic Ivan Ting had shared the Choosing Wisely experience Brazil and the Philippines, respectively [1, 2].

Led by Dr Fidel Rubagumya, the CWA core working group met in 2020 and came up with ten practices that should be avoided in cancer care in Africa [3]. Building upon the already published Choosing Wisely Canada [4], India [5] and United States of America [6, 7] guidelines, the CWA recommendations were developed [3]. Recommendations would ensure delivery of the best cancer care at the lowest costs, hence avoidance of financial toxicity to patients and their families translating to responsible stewardship of resources. For each recommendation, specific criteria were considered as each harmful practice was deliberated and these included: evidence of low value/harm; high frequency of use; costs (including opportunity cost); clarity on wording of each practice item; relevance to the African cancer context and feasibility of future measurement activity. Ultimately, ten low value or harmful practices that should be avoided in cancer practice in Africa were recommended and published in the Journal of Clinical Oncology, Global Oncology in 2020 [8]. During the Senegal CWA conference, Dr Rubagumya shared with the audience how CWA was developed and the importance of value-based cancer care in Africa. He stressed the known fact that African governments do not allocate adequate funding for healthcare in their respective budgets. Therefore it is the duty of other stakeholders to advocate for practices that are affordable and clinically beneficial to patients to ensure equitable use of available resources.

## Workshops on Choosing Wisely by oncology specialty

Guided by the ten low value or harmful practices to be avoided in cancer care in Africa, six presentations focused on harmful practices specific to each oncology specialty were shared. Professor Sidy Ka (Cheikh Anta Diop University, Senegal) led the Choosing Wisely in surgical oncology discussion, highlighting that most practices specific to surgery were adopted from established guidelines from Canada, India and the USA and because of limited resources, these are a challenge to implement [4–6]. He went on to acknowledge the importance of establishing multidisciplinary teams in Africa as a key to best practice and expanding surgical oncology training in Africa. Dr Omar Abdihamid (Garissa Cancer Center, Kenya) shared a comprehensive talk on Choosing Wisely in Haemato-oncology. He emphasised the importance of using single-agent chemotherapy instead of combination therapy when indicated [9]. He proceeded to share perspectives on the appropriate utilisation of targeted therapy use and de-escalation therapy in geriatric oncology [10]. He discussed anti-emetic avoidance in patients starting low emetogenic chemotherapy [9], avoidance of routine pre-operative haemostatic testing in healthy patients, avoiding transfusion in asymptomatic patients or those with chronic anaemia and avoiding anticoagulation for more than 3 months in patients experiencing a first venous thromboembolic event in the setting of major transient risk factors for venous thromboembolism [11, 12]. He proceeded to share an insightful real-world approach on adopting Choosing Wisely guided by practice setting at Garissa Cancer Center, a rural community comprehensive cancer centre in Kenya where he practices. He cited head and neck cancers and oesophageal cancers to be the most common cancers in his practice in which most patients present with locally advanced cancer. The current management approach for most patients is concurrent chemo-radiotherapy (98%) and there is low uptake of oesophagectomy after noticing high morbidity post-surgery. Therefore, oesophagectomy is not recommended at his centre unless a patient can travel to a high-volume centre. These real practice experiences are encouraged to be shared as they will provide guidance in subsequent meetings to identify where support is needed to guide optimum care and as recommendations are reviewed and further aligned to the African setting.

Dr Melinda Mushonga (University of Toronto, Canada and University of Zimbabwe) gave a talk on Choosing Wisely in radiation oncology. The discussion gave an overview of practices that align more on radiation therapy with a focus on adoption of hypofractionation in situations that have a solid clinical evidence base [13, 14] and in the palliative setting [15–17]. She further discussed treatment de-escalation [18] and in some cases omission of radiotherapy if not clinically indicated or if benefit is not meaningful from a clinical perspective [10]. Dr Mushonga emphasised the need for radiation oncology aligned clinical trials in Africa to build the evidence base for patients being managed in low-resource centres. Her presentation was complimented by a talk on breast intraoperative radiotherapy and ultra-hypofractionation delivered by Dr Sofia Rivera (Gustave Rousy Cancer Campus Grand Paris, France). Reference was made to the Targit A trial [19] and Fast Forward trial [20] which both showed equivalence in clinical outcomes to multifractionated radiotherapy in appropriately selected patients.

Choosing Wisely in palliative care was led by Dr Christian Ntizimira (African Center for Research on End-of-Life Care, Rwanda). The importance of palliative care incorporation in cancer care was reinforced and early intervention encouraged including its integration with social-cultural aspects to humanise cancer care in Africa. The diversity of population, the perception of life, care, death and dying must be taken into consideration when a palliative care programme is implemented in cancer centre [21]. Training and establishment of decentralised palliative care services to meet community needs was recommended emphasising that palliative care is not an optional service, not an add-on, luxury or an afterthought but a vital component of compassionate cancer care. He concluded ‘When access to radiotherapy, screening, and chemotherapy are discussed [22] – all of which are critical – access to palliative care in equal amount should be discussed and with the same passion!’

Dr Verna Vanderpuye (Korle Bu Teaching Hospital, Ghana), one of the pioneers of the CWA core working group since inception, gave an insightful talk on navigating evidence-based care with limited resources in Africa. She emphasised the need for well-designed clinical trials that can be implemented in Africa which align with available resources. She highlighted the current challenge of having most if not all practice changing trials being designed and conducted in settings that do not mimic the current practice setting in Africa [23]. She gave an important example in cervical cancer, the most common cancer in most regions in Africa. The current international standard approach of management is guided by the EMBRACE protocol which includes MRI-guided brachytherapy [24]. There is not a single cancer centre in Africa offering MRI-guided brachytherapy as part of cervical cancer management yet the regions with the highest prevalence. There are very few trials in radiation oncology from low middle income countries, Africa included [25]. This therefore highlights the need to support radiation oncology clinical trials at least in disease sites that are common in Africa.

Dr Gad Murenzi (Einstein-Rwanda Research and Capacity Building Program, Research for Development (RD Rwanda) and Rwanda Military Hospital) presented on ‘Choosing wisely in implementing prevention, screening and Early detection programs in Africa’ where he highlighted the need to follow guidelines including CWA recommendations and put up a food-for-thought question; ‘*Could not following evidence-based guidelines and recommendation be a low value practice as part of choosing wisely?’* He then continued to underscore some low-hanging fruits for prevention and screening that could be implemented in resource-limited settings, such as HPV vaccination, HPV testing and self-specimen collection for cervical cancer prevention, self-breast examination, foecal occult blood test for colorectal cancer among others. In addition, he emphasised the need to adopt to new recommendations guided by evidence particularly in Africa where it usually takes a while with an illustration of a single-dose HPV vaccine which could be both feasible and cost-effective. Finally, he highlighted challenges with laboratory services especially pathology and its advanced/ancillary testing such as molecular testing. He discussed the need for research and innovation for both locally derived data and home-grown solutions.

## Implementing Choosing Wisely in cancer care

A follow-up 2 years post publication of CWA was conducted by Rubagumya *et al* [26]. The survey completed by the workforce in oncology captured information on awareness of the CWA, agreement with recommendations and concordance with clinical practice. There was strong agreement in awareness of the recommendations and agreement with CWA recommendations across all ten recommendations. Most stated that they ‘always’ or ‘often’ follow CWA recommendations, but lowest adherence was reported for radiation oncology specific recommendations. For example, use of shorter courses of radiation when appropriate (67%) and discussing active surveillance with patients with low-risk prostate cancer (70%). Several participants reported that implementing CWA recommendations would be relatively straightforward, but the challenging recommendations to implement were shorter courses of radiation when appropriate (55%), considering neoadjuvant therapy instead of upfront surgery (51%) and multidisciplinary input for curable cancers (50%) [26]. These concerns were re-affirmed in the series of presentations as highlighted earlier. There was a stimulating discussion in the question-and-answer segment on why there was reduced uptake of hypofractionation and neoadjuvant therapy overall. It was suggested the current fractionation- based payment model does not promote uptake of hypofractionation due to the potential reduced income. Lack of clinical evidence on hypofractionation specifically emanating from Africa is of concern to a number of clinicians and lack of tumor board discussions were additional possible reasons for the reduced uptake of hypofractionation. These discussion points are food for thought on some aspects in clinical care that need to be supported for implementation of Choosing Wisely recommendations. These include at policy level, reviewing reimbursement strategies [27–29] as suggested in the published literature, supporting clinical trials in Africa to increase the evidence base in Africa and innovative ways of conducting tumour boards without increasing workload or delaying care.

## The role of the workforce in value in cancer care

Professor Nazik Hammad (Queen’s University, Canada) further discussed the role of the workforce in implementing Choosing Wisely in cancer care. She reaffirmed that high-value care is providing the highest quality care at the lowest cost requiring avoiding overuse, underuse and misuse and practicing evidence-based care coupled with adequate communication between providers, i.e. the workforce, and patients and their families and also among providers. As such the workforce plays a vital role in value-based care and implementing Choosing Wisely initiatives. She acknowledged the workforce may fail to provide evidence-based care because of a lack of knowledge or familiarity with practice guidelines. This in turn could be due to inadequate training, inadequate access to learning resources, inadequate self-directed and life-long learning skills and/or a lack of system-level interventions and monitoring of medical competencies. Secondly, the workface can fail to provide evidence-based care due to high-volume workload including very busy clinics, administrative and teaching loads, unregulated dual practice, inadequate quality monitoring and metrics for guideline concordance, a lack of motivation because of inadequate compensation and unfavourable workplace conditions and inadequate training in communication, professionalism and humanism. Therefore, ttransforming and upscaling health professional education (HPE) is essential in addressing the gaps in the workforce role in providing value-based cancer care. This includes producing adequate number (quantity) of cancer health professionals who will have time to provide adequate care and communication with patients and their families. A second dimension of transforming HPE was proposed, and this involves equipping the workforce with competencies related to medical knowledge and competencies related to leadership and stewardship of resources. The third dimension is training health professionals who can function in the context for which they have been trained and who can address the health needs of their own population. She highlighted the roadmap to getting there is through changing the way we train oncology workforce and by utilising competency-based medical education which encompasses in addition to medical knowledge, competencies in communication, critical thinking, ethical deliberation and stewardship of resources [30]. Investment in training programme development and evaluation and faculty development is pivotal. The global oncology community needs to come together to share best practices in education and to harness the power of IT. She applauded the oncology training in Africa as its design makes the residents in training more cognisant of cost of care and affordability as they directly participate in the care of patients independently much earlier in their training. She encouraged clinicians in academia to harness this established training setup in conceptualising the Choosing Wisely mentality as they transition to independent practice upon completing training.

## Conclusion

The inaugural CWA conference was a major positive initiative aimed to raise awareness about practices that are of low value, costly and sometimes harmful to patients and that should be avoided in Africa. The programme acquainted participants with the overall development of CWA and was a platform to discuss evidence available which aligns to Choosing Wisely initiatives making it a success. It, however, highlighted the gaps and challenges in implementing some of the initiatives therefore providing recommendations which include more focused educational interventions that target the different specialities in oncology. Next steps include, firstly, regional meetings in Africa to assist in dissemination of Choosing Wisely initiatives a platform that will promote more adoption and uptake of the recommendations. Second, measurement of CWA impact in clinical practice. Third, development of region aligned recommendations which fit the diverse practice environment and local evidence. Fourth, development of speciality-based recommendations and finally updating the initial list.

## Conflicts of interest

None.

## Funding

The meeting was funded by *e*cancer.
